# Differential impact of heat and hypoxia on dynamic oxygen uptake and deoxyhemoglobin parameters during incremental exhaustive exercise

**DOI:** 10.3389/fphys.2023.1247659

**Published:** 2024-01-08

**Authors:** Zhizhong Geng, Jinhao Wang, Guohuan Cao, Chenhao Tan, Longji Li, Jun Qiu

**Affiliations:** ^1^ School of Sports and Health, Shanghai University of Sport, Shanghai, China; ^2^ Shanghai Research Institute of Sports Science, Shanghai, China

**Keywords:** high temperature and humidity environment, hypoxic environment, incremental exhaustive exercise, deoxyhemoglobin dynamics, oxygen uptake dynamics

## Abstract

**Purpose:** This study aims to explore the relationship between the dynamic changes in oxygen uptake ( $\dot{V}O_{2}$ ) and deoxyhemoglobin (HHb) and peripheral fatigue in athletes during incremental exhaustive exercise under different environmental conditions, including high temperature and humidity environment, hypoxic environment, and normal conditions.

**Methods:** 12 male modern pentathlon athletes were recruited and performed incremental exhaustive exercise in three different environments: normal condition (23°C, 45%RH, FiO_2_ = 21.0%, CON), high temperature and humidity environment (35°C, 70%RH, FiO_2_ = 21.0%, HOT), and hypoxic environment (23°C, 45%RH, FiO_2_ = 15.6%, HYP). Gas metabolism data of the athletes were collected, and muscle oxygen saturation (SmO_2_) and total hemoglobin content in the vastus lateralis muscles (VL) were measured to calculate the deoxyhemoglobin content. Linear and nonlinear function models were used to fit the characteristic parameters of $\dot{V}O_{2}$ and HHb changes.

**Results:** The results showed that compared to the CON, $\dot{V}O_{2}$ , $\dot{V}{\text{CO}}_{2}$ , and exercise time were decreased in the HOT and HYP (*p* < 0.05). $\Delta E_{\dot{V}O2}$ and OUES were reduced in the HOT and HYP compared to the CON (*p* < 0.05). The Gas exchange threshold in the CON corresponded to higher $\dot{V}O_{2}$ than in the HYP and HOT (*p* < 0.05). ${\Delta E}_{\dot{V}O2‐1}$ was reduced in the HOT compared to the HYP (*p* < 0.05). ΔE_HHb_ was higher in the HOT compared to the CON (*p* < 0.05). ΔE_HHb-1_ was increased in the HYP compared to the CON (*p* < 0.05). There was a negative correlation between ΔE_HHb_ and corresponding $\dot{V}O_{2\max}$ in the HOT (r = −0.655, *p* < 0.05), and a negative correlation between ΔE_HHb-1_ and corresponding $\dot{V}O_{2\max}$ in the HYP (r = −0.606, *p* < 0.05).

**Conclusion:** Incremental exhaustive exercise in hypoxic environment and high temperature and humidity environments inhibits gas exchange and oxygen supply to skeletal muscle tissue in athletes. For athletes, the accelerated deoxygenation response of skeletal muscles during incremental exhaustive exercise in high temperature and humidity environments, as well as the excessive deoxygenation response before BP of deoxyhemoglobin in hypoxic environment, may be contributing factors to peripheral fatigue under different environmental conditions.

## 1 Introduction

Competitive sports athletes frequently encounter various challenging training and competition environments, such as high temperature, high humidity, and hypoxic conditions. However, when the human body operates in these environments, it often experiences a decline in physical performance and an accelerated onset of exercise fatigue, among other negative effects (Osawa et al., 2017; Jung et al., 2021).

Hypoxia exposure, in comparison to normal environmental conditions, can restrict respiratory function and affect gas exchange in the human body. It can lead to a decrease in arterial oxygen saturation (SpO_2_), capillary oxygen partial pressure, and ultimately limit oxygen supply to peripheral tissues (Twomey et al., 2017). Numerous studies have consistently demonstrated that hypoxia exposure leads to a decrease in exercise time, maximal oxygen uptake ( $\dot{V}O_{2\max}$ ), and peak output power among athletes during incremental exhaustive exercise (Lawler et al., 1988; González-Alonso and Calbet, 2003; Subudhi et al., 2007). Furthermore, it induces a leftward shift in the Gas Exchange Threshold (GET) and Respiratory Compensation Point (RCP) (Zerbini et al., 2013; Bowen et al., 2016). Furthermore, research focusing on the impact of hypoxia exposure have demonstrated that during incremental exhaustive exercise, break point (BP) of active muscle deoxy-hemoglobin (HHb) undergoes a leftward shift under hypoxic conditions (Azevedo et al., 2020a). Additionally, when athletes engage in physical activities in high-temperature and humid environments, heat exposure can contribute to the acceleration of peripheral fatigue by influencing gas exchange and oxygen transport in skeletal muscle. The challenging conditions in such environments often lead to disruptions in heat dissipation, elevated core temperature (Tc), excessive dehydration, and impaired functional regulation of the cardiovascular, central nervous, and musculoskeletal systems (Périard et al., 2013; Yamaguchi et al., 2021). Consequently, these factors can restrict the capacity for oxygen transport and utilization in skeletal muscle tissue. As a result, athletes experience a leftward shift in the GET, along with a decrease in $\dot{V}O_{2\max}$ and exercise time, when performing incremental exhaustive exercise under high-temperature and humid conditions (Tatterson et al., 2000; Sawka et al., 2011).

The dynamic characteristics of Oxygen Uptake ( $\dot{V}O_{2}$ ) and HHb during incremental exercise, particularly in special environments such as high temperature and humidity, and hypoxic environments, have not been extensively studied. Previous research has shown that $\dot{V}O_{2}$ exhibits a linear increase with exercise intensity, while HHb in skeletal muscle demonstrates a bilinear increase with exercise load (Vieth, 1989; Spencer et al., 2012). However, the specific differences in dynamic parameters between $\dot{V}O_{2}$ and HHb during incremental exhaustive exercise under these special environments remain unclear. Exploring the dynamic changes of HHb in body gas exchange and skeletal muscle microcirculation during exercise can provide valuable insights into the mechanisms underlying premature peripheral fatigue in these environments.

Therefore, it is crucial to investigate the characteristics of oxygen supply in peripheral tissues and body gas exchange among athletes in high temperature, high humidity, and hypoxic environments. In our study, we utilized Near-Infrared Spectroscopy (NIRS) as a non-invasive method to assess oxygen levels in micro vessels. By comparing the characteristic parameters of oxygen uptake kinetics and deoxyhemoglobin kinetics in athletes during incremental exhaustive exercise under high temperature and high humidity, hypoxic, and normal environments, we aim to explore the influence of these different special environments on the dynamic changes of $\dot{V}O_{2}$ and Muscle Oxygen Saturation (SmO_2_) during exercise.

## 2 Materials and methods

### 2.1 Subject

Twelve male modern pentathletes (age = 17.91 ± 2.94 years; height = 1.81 ± 0.06 m; body mass = 70.95 ± 8.38 kg; body mass index = 21.69 ± 1.83 kg/m^2^; training years = 5.33 ± 2.92 years) participated in the study, with no dropouts recorded. Prior to the study, the participants were provided with detailed information about the experimental procedures and the purpose of the study. They were also informed about the potential risks and benefits associated with their participation. Informed consent forms were provided to the participants, and they were given sufficient time to review and understand the information before signing the consent forms. For athletes under the age of 18, approval was sought from the athlete’s legal guardian or close relative. The study specifically involved athletes from the modern pentathlon team of Shanghai Chongming Sports Training Base, who voluntarily agreed to participate in the research. Confidentiality and anonymity of the participants’ personal information were ensured throughout the study.

### 2.2 Experimental design

#### 2.2.1 Environmental parameters

This study was conducted in the Special Environment Laboratory at the Shanghai Institute of Sports Science. Three different exercise environments were set up: high temperature and humidity with normoxia (HOT), normal temperature and humidity with hypoxia (HYP), and normal temperature and humidity with normoxia (CON). The environmental parameters were as follows: High temperature and humidity with normoxia: 35°C, 70% relative humidity (RH), and fractional inspired oxygen concentration (FiO_2_) = 21.0%. Normal temperature and humidity with hypoxia: 23°C, 45% RH, and FiO_2_ = 15.3%. Normal temperature and humidity with normoxia: 23°C, 45% RH, and FiO_2_ = 21.0%.

#### 2.2.2 Experimental procedure

The athletes performed incremental exercise tests in the three different environments. The CPET test was administered by researchers following a standardized procedure tailored to the characteristics of modern pentathlon athletes, conducted on a treadmill. Prior to the experiment, athletes engaged in a 10-min standardized warm-up and stretching routine, donning necessary equipment such as breathing masks and heart rate monitors before commencing the formal test.

The initial load was set at 8 km/h with 0% incline. Subsequently, the speed was increased by 1 km/h every 1 min while maintaining the incline. When the treadmill speed reached 18 km/h, the speed was no longer increased, but instead, the incline was increased by 1% every 1 min. There were no breaks between the levels. Gas metabolism, heart rate, and other relevant data were continuously collected without intervals between levels. The test termination criteria included various indicators such as dyspnea, cyanosis, dizziness, tinnitus, nausea, chest pain, extreme fatigue, painful expression, pale face, and body shaking. Additionally, the test could be terminated if the participant’s heart rate reaches the expected maximum heart rate, if the subject requests to stop the test, if the Borg Rating of Perceived Exertion (RPE) reaches or exceeds 17, or if the participant is unable to maintain the required speed. In any of these situations, the test was immediately stopped to ensure the safety and wellbeing of the participants.

We implemented a randomized crossover design, conducting participant tests in diverse environments at the same time of day on days 1, 3, and 5, with a 48-h interval between each session. Before each test, the athletes’ physiological parameters were checked to ensure their good health and normal physical function.

#### 2.2.3 Cardiopulmonary responses

Gas metabolism data during the incremental exercise tests were collected using the COSMED gas analyzer (COSMED Quark PFT ergo, OMNIA CPET, Italy). The following parameters were obtained: $\dot{V}O_{2}$ , carbon dioxide production ( $\dot{V}{\text{CO}}_{2}$ ), respiratory exchange ratio (RER), respiratory rate (RR), tidal volume ( $\dot{V}T$ ), and minute ventilation ( $\dot{V}E$ ). Heart rate (HR) changes during exercise were recorded using a heart rate monitor (Polar RS800CX, Polar Electro, Kempele, Finland), and Tc was recorded using a core temperature capsule (e-Celsius^®^, BMedical Pty LTb, BodyCap, Australia). Immediately after exercise, SpO_2_ was measured using a finger pulse oximeter (YX302, Yuwell Medical, China), and fingertip blood samples were collected to assess the maximal blood lactate (Bla) concentration.

#### 2.2.4 Tissue oxygenation

The NIRS signal in human tissues predominantly originates from the absorption of light by hemoglobin (Hb) in arterioles, capillaries, and venules. In muscle tissue, myoglobin (Mb) contributes approximately 10% to the NIRS light absorption signal (Seiyama et al., 1988; Chance et al., 1992). However, due to the overlap of Mb and Hb absorption spectra, they are indistinguishable in NIR spectra. Near-infrared spectral signals primarily indicate the availability of oxygen in tissue microcirculation (Boushel et al., 2001). Furthermore, serving as a monitoring instrument, the MOXY (Moxy Muscle Oxygen Sensor, Hutchinson, Minnesota, United States) has demonstrated reliability in monitoring SmO2 and THb (Crum et al., 2017). Muscle oxygen saturation data were collected using the MOXY near-infrared spectroscopy (NIRS) device. The device utilizes NIRS to measure the concentrations of oxyhemoglobin (O_2_Hb), deoxyhemoglobin (HHb), and total hemoglobin (THb) in the muscle tissue during incremental exercise tests and recovery after exercise. The data were sampled at a frequency of 1 Hz. The NIRS probe was placed on the skin surface above the vastus lateralis muscles (VL) belly of the dominant lower limb and securely covered to prevent light interference (Yamaguchi et al., 2021). SmO_2_ was calculated based on a modified form of the Beer-Lambert law (Saitoh et al., 2010).

### 2.3 Data analyses

#### 2.3.1 Oxygen uptake kinetics

In order to analyze the data in our study, we applied a smoothing technique to $\dot{V}O_{2}$ , VE, RF and VT for a 10-s interval during the incremental exhaustive exercise. Given the variations in athletes’ exercise duration across the three environments, we calculated the mean value of $\dot{V}O_{2}$ during the last 30 s of each stage load within the initial 10 min of the exercise test for further analysis. Additionally, average values of VT, RF, and VE were calculated every 30 s during exercise to evaluate the influence of environmental factors before reaching GET. Recognizing variations in individual GET, we specifically analyzed data collected before 270s. To further analyze the data, we employed Origin software (OriginLab, 2018 Pro, United States) to fit the entire motion process data using two formulas. The first Formula (1) represents a linear fit between $\dot{V}O_{2}$ and time during the exercise, and the second Formula (2) represents a logarithmic function fit between $\dot{V}O_{2}$ and VE during the exercise. This fitting process allows us to examine the relationship between $\dot{V}O_{2}$ and time, as well as $\dot{V}O_{2}$ and VE throughout the exercise protocol.

To determine the GET during the exercise, we utilized the V-slope method (Beaver et al., 1986). The inflection point of the VE/ $\dot{V}{\text{CO}}_{2}$ relationship was employed to determine RCP (Whipp et al., 1989). The GET served as a pivotal point in the exercise protocol, dividing the incremental exhaustive exercise into two stages for bilinear fitting. The bilinear fitting was performed using Formula (1) to analyze the relationship between $\dot{V}O_{2}$ and time. Model parameter estimation was carried out using linear least squares regression analysis. The slopes of the calculated linear fitting equations were designated as $△E_{\dot{V}O2‐1}$ and $△E_{\dot{V}O2‐2}$ , representing the two stages of the exercise from the start to GET and from GET to exhaustion, respectively.

$$\dot{V}O_{2\left(t\right)}=\dot{V}O_{2baseline}+{△E}_{VO2}\cdot t$$ (1)

$$\dot{V}O_{2\left(VE\right)}=\dot{V}O_{2baseline}+OUES\cdot \log10\left(VE\right)$$ (2)

Where $\dot{V}O_{2\left(t\right)}$ is the oxygen uptake at time t, $\dot{V}O_{2\text{baseline}}$ is the oxygen uptake at baseline, $△E_{\dot{V}O2}$ is the linear fitting slope, $\dot{V}O_{2\left(\text{VE}\right)}$ is the oxygen uptake corresponding to VE. OUES is the Oxygen Uptake efficiency Slope, that is the rate of change of $\text{VE}/\dot{V}O_{2}$ .

#### 2.3.2 Deoxyhemoglobin kinetics

In the incremental exhaustive exercise, the NIRS measurements of SmO_2_, [HHb], and [THb] were smoothed using a 10-s interval. To account for variations in athletes’ exercise time in different environments, the mean values of SmO_2_, [HHb], and [THb] were calculated for the last 30 s of each stage load during the initial 10 min of the exercise test.

The software Origin was utilized for analyzing the dynamic changes of [HHb] measured by NIRS during the exercise test and to calculate the dynamic parameters of [HHb] over the course of exercise. Formula (3) was employed to perform a linear fit between [HHb] and time during exercise. Additionally, the exercise duration was divided into two sections based on the BP, namely, from the start of exercise to BP and from BP to the point of exhaustion. Bilinear fitting using Formula (3) was applied to these two sections. The model parameters were estimated using linear least squares regression analysis, and the slopes of the linear fitting equations were represented as △E_HHb-1_ and △E_HHb-2_, respectively (Spencer et al., 2012).

To assess the dynamic relationship between oxygen uptake and oxygen utilization during exercise, $△\left[\text{HHb}\right]/△\dot{V}O_{2}$ was normalized for both time and amplitude. The baseline value was assigned 0%, while the steady-state value was assigned 100%. The standardized $\dot{V}O_{2}$ was shifted left by 20 s to align with the [HHb] (Murias et al., 2014), after which the ratio of the two was calculated to obtain the $△\left[\text{HHb}\right]/△\dot{V}O_{2}$ curve (Boone et al., 2015).

$${\left[HHb\right]}_{\left(t\right)}={\left[HHb\right]}_{baseline}+{△E}_{HHb}\cdot t$$ (3)

Where [HHb]_(t)_ is the deoxyhemoglobin value at time t, [HHb]_baseline_ is the baseline deoxy hemoglobin value, and △E_HHb_ is the linear fitting slope.

### 2.4 Statistical analysis

The statistical analysis of the experimental data was conducted using SPSS 21.0 software (IBM SPSS Statistics 21, IBM Cooperation, Chicago, IL). The data were presented as mean ± standard deviation (Mean ± SD). For normally distributed data with homogeneous variance, the parameter test was chosen. ANOVA with Repeated Measures was used to analyze the experimental data, and the Bonferroni method was employed for *post hoc* comparisons between groups to identify any significant differences. If the data did not meet the assumptions of normal distribution or homogeneity of variance, non-parametric tests were used instead. The goodness of fit of the regression model coefficients was evaluated using regression analysis and the Coefficient of Determination (*R* ^2^). Additionally, the Pearson correlation coefficient (r) was utilized to analyze the correlation between △E_HHb-1_, △E_HHb_, and $\dot{V}O_{2\max}$ during exercise under various environmental conditions. The confidence interval was set at 95%, and the significance level was *α* = 0.05.

## 3 Results

### 3.1 Gas metabolism

The results of the study showed that compared to the CON, athletes in the HOT and HYP exhibited reductions in $\dot{V}O_{2}$ at exhaustion (F(2,22) = 6.832, *p* = 0.005, η_p_^2^ = 0.383, CON vs. HOT *p* = 0.012, 95%CI [149.652, 397.937]; CON vs. HYP *p* = 0.038, 95%CI [-47.983, 2001.856]), as well as relative $\dot{V}O_{2}$ (F (2, 22) = 17.161, *p* < 0.001, η_p_^2^ = 0.609, CON vs. HOT *p* = 0.012, 95%CI [1.231, 20.459]; CON vs. HYP *p* = 0.002 [95%CI 4.282, 23.423]; HOT vs. HYP *p* = 0.012 95%CI [0.358, 5.657]). They also showed decreases in $\dot{V}{\text{CO}}_{2}$ (F (2, 22) = 6.288, *p* = 0.007, η_p_^2^ = 0.364, CON vs. HOT *p* = 0.058, 95%CI [-160.213, 2024.822]; CON vs. HYP *p* = 0.010 95%CI [-291.809, 2021.921]) and relative $\dot{V}{\text{CO}}_{2}$ (F (2, 22) = 13.938, *p* = 0.003, η_p_^2^ = 0.559, CON vs. HOT *p* = 0.003, 95%CI [3.540, 23.322]; CON vs. HYP *p* = 0.013, 95%CI [1.345, 24.095]), as well as a decrease in exercise time (F (2, 22) = 10.158, *p* = 0.001, η_p_^2^ = 0.480, CON vs. HOT *p* = 0.055, 95%CI [-0.253, 3.421 ]; CON vs. HYP *p* = 0.011, 95%CI [95%CI 0.279, 3.985]). Compared to the HOT, the HYP exhibited a increase in RER (F (1.899, 20.892) = 5.396, *p* = 0.014, η_p_^2^ = 0.329, HOT vs. HYP *p* = 0.017, 95%CI [0.004, 0.144]) and an elevated Bla (F (1.583, 17.416) = 7.383, *p* = 0.007, η_p_^2^ = 0.402, HOT vs. HYP *p* = 0.025, 95%CI [0.015, 9.402 ]). There was not a reduction in Tc in Hyp and CON, but rather an increase in Tc in HOT conditions compared to the others (F (1.842, 20.259) = 14.663, *p* < 0.001, η_p_^2^ = 0.571, CON vs. HOT *p* < 0.001, 95%CI [0.230, 0.838]; HOT vs. HYP *p* = 0.012, 95%CI [0.500, 0.813]). Additionally, we would expect a lower SpO_2_ in HYP compared to CON and HOT conditions (F (2, 22) = 49.023, *p* < 0.001, η_p_^2^ = 0.817, HYP vs. HOT *p* < 0.001, 95%CI [6.062, 17.104]; CON vs. HYP *p* < 0.001, 95%CI [6.880, 15.453]), are shown in Table 1.

**TABLE 1:** Gas metabolism parameters at exhaustion during incremental exhaustive exercise in different environments.

Variables	HOT	HYP	CON	*p*
RF (cpm)	57.88 ± 7.00	59.00 ± 12.09	58.79 ± 11.21	0.916
VT (L)	2.59 ± 0.55	2.63 ± 0.61	2.7 ± 0.76	0.918
VE (L/min)	148.81 ± 29.17	151.49 ± 26.69	153.38 ± 24.4	0.916
$\dot{V}O_{2}$ (mL/min)	3791.91 ± 613.39^#^	3581.09 ± 574.27^#^	4543.11 ± 807.96	0.005^*^
$\dot{V}O_{2}$ (ml/min·kg)	53.53 ± 5.04^#^	50.52 ± 4.54^#^	64.37 ± 7.76	<0.001^*^
$\dot{V}{\text{CO}}_{2}$ (mL/min)	3943.74 ± 536.64^#^	4010.99 ± 707.4^#^	4876.05 ± 792.23	0.007^*^
$\dot{V}{\text{CO}}_{2}$ (ml/min·kg)	55.79 ± 4.59^#^	56.5 ± 5.83^#^	69.23 ± 8.22	0.003^*^
HR (bpm)	193.45 ± 9.3	191 ± 8.67	190.91 ± 9.48	0.763
RER	1.04 ± 0.05^&^	1.12 ± 0.05	1.08 ± 0.06	0.014^*^
Bla (mmol/L)	11.42 ± 2.56^&^	16.13 ± 4.75	13.43 ± 2.93	0.007^*^
Tc (°C)	39.06 ± 0.21^&#^	38.52 ± 0.31	38.63 ± 0.36	<0.001^*^
SpO_2_ (%)	97.83 ± 1.27^&^	86.25 ± 5.26^#^	97.42 ± 1.78	<0.001^*^
Exercise time (min)	13.14 ± 1.42^#^	12.59 ± 1.59^#^	14.73 ± 1.17	0.001^*^

* Indicates statistical of intergroup differences (*p* < 0.05), # indicates difference compared to the CON (*p* < 0.05), and and indicates difference compared to the HYP (*p* < 0.05).

**FIGURE 1:**
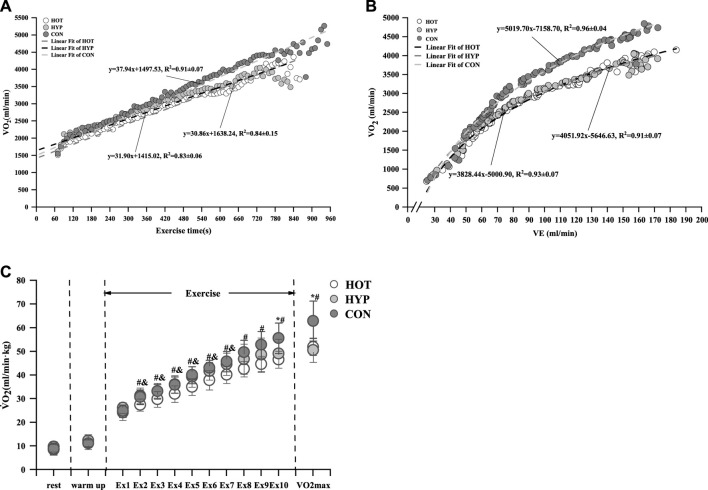
Linear fit of $\dot{V}O_{2}$ **(A)** and logarithmic fit of $\dot{V}O_{2}$ and VE **(B)** and changes in $\dot{V}O_{2}$ **(C)** during incremental exhaustive exercise under different conditions. Note: * indicates a difference between the CON and HYP (*p* < 0.05), # indicates a difference between the CON and HOT (*p* < 0.05), and & indicates a difference between the HOT and HYP (*p* < 0.05).

After linear regression analysis of $\dot{V}O_{2}$ and exercise time in each group, it was found that the HOT had *R* ^2^ = 0.84 ± 0.15 (*p* < 0.01), the HYP had *R* ^2^ = 0.83 ± 0.06 (*p* < 0.01), and the CON had *R* ^2^ = 0.91 ± 0.07 (*p* < 0.01) as shown in Figure 1A. Compared to the CON, both the HOT and HYP showed reduced $△E_{\dot{V}O2}$ (F (1.747, 19.217) = 4.837, *p* = 0.023, η_p_^2^ = 0.305, CON vs. HOT *p* = 0.029, 95%CI [0.880, 13.264]; CON vs. HYP *p* = 0.034, 95%CI [0.556, 12.019]). Additionally, by comparing the nonlinear logarithmic regression analysis of $\dot{V}O_{2}$ and VE during exercise, it was found that the HOT had *R* ^2^ = 0.91 ± 0.07 (*p* < 0.01), the HYP had *R* ^2^ = 0.93 ± 0.07 (*p* < 0.01), and the CON had *R* ^2^ = 0.96 ± 0.04 (*p* < 0.01), as shown in Figure 1B. Compared to the CON, both the HYP and HOT showed reduced OUES (F (2, 22) = 8.333, *p* = 0.002, η_p_^2^ = 0.431, CON vs. HOT *p* = 0.024, 95%CI [162.155, 1873.418]; CON vs. HYP *p* = 0.008, [95%CI 385.768, 1996.751]). Furthermore, it was found in this study that the exercise time corresponding to GET was shorter in the HYP compared to the CON(F (1.666, 18.330) = 7.491, *p* = 0.003, η_p_^2^ = 0. 405, CON vs. HYP *p* = 0.015, 95%CI [0.303, 2.863]). The $\dot{V}O_{2}$ at GET was higher in the CON compared to the HOT (F (1.766, 19.421) = 7.285, *p* = 0.004, η_p_^2^ = 0. 398, CON vs. HOT *p* = 0.004, 95%CI [165.044, 799.057]), and the relative $\dot{V}O_{2}$ at GET was higher in the CON compared to the HYP and HOT (F (1.743, 19.170) = 9.805, *p* = 0.002, η_p_^2^ = 0.471, CON vs. HOT *p* = 0.001, 95%CI [3.484, 11.090]; CON vs. HYP *p* = 0.049, 95%CI [0.027, 10.807]). Using GET as a reference point, linear regression analysis was performed on $\dot{V}O_{2}$ and exercise time before and after GET in each group. The results showed that the HOT had $R_{\dot{V}O2‐1}^{2}$ = 0.84 ± 0.04 (*p* < 0.01), $R_{\dot{V}O2‐2}^{2}$ = 0.89 ± 0.08 (*p* < 0.01). The HYP had R $R_{\dot{V}O2‐1}^{2}$ ^2^ = 0.81 ± 0.08 (*p* < 0.01), $R_{\dot{V}O2‐2}^{2}$ = 0.87 ± 0.08 (*p* < 0.01). The CON had $R_{\dot{V}O2‐1}^{2}$ = 0.86 ± 0.03 (*p* < 0.01), $R_{\dot{V}O2‐2}^{2}$ = 0.91 ± 0.08 (*p* < 0.01). Compared to the HYP, both the HOT and CON showed reduced $△E_{\dot{V}O2‐1}$ values (F (2, 22) = 8.181, *p* = 0.002, η_p_^2^ = 0.427, HYP vs. HOT *p* = 0.002, 95%CI [10.096, 29.136]; CON vs. HYP *p* = 0.030, 95%CI [2.052, 32.785]) as shown in Table 2.

**TABLE 2:** Differences in kinetic parameters of $\dot{V}O_{2}$ during incremental exhaustive exercise in different environments.

Variables	HOT	HYP	CON	*p*
$\dot{V}O_{2}$ @GET (mL/min)	2643.74 ± 436.69^#^	2770.26 ± 390.49	3125.79 ± 390.71	0.004^*^
$\dot{V}O_{2}$ @RCP (mL/min)	3131.59 ± 424.58^#^	3053.44 ± 539.85^#^	3642.27 ± 310.47	0.004^*^
$\dot{V}O_{2}$ @GET (ml/min·kg)	37.4 ± 4.62^#^	39.26 ± 4.70^#^	44.64 ± 4.42	0.002^*^
$\dot{V}O_{2}$ @RCP (ml/min·kg)	44.37 ± 4.47^#^	43.18 ± 5.70^#^	52.22 ± 5.02	<0.001^*^
Time@GET (min)	6.25 ± 1.02	6.13 ± 0.83^#^	7.67 ± 1.38	0.003^*^
Time@RCP (min)	8.79 ± 1.43^&^	7.35 ± 1.08^#^	9.96 ± 1.33	<0.001^*^
$△E_{\dot{V}O2}$ (ml/min·s)	30.86 ± 6.10^#^	31.90 ± 5.46^#^	37.94 ± 6.51	0.023^*^
$△E_{\dot{V}O2‐1}$ (ml/min·s)	47.08 ± 6.97^&^	66.69 ± 18.35	49.27 ± 9.83	0.002^*^
△E V˙O2‐2 (ml/min·s)	34.33 ± 22.00	27.96 ± 17.68	35.19 ± 14.77	0.580
OUES	4051.92 ± 818.58	3828.44 ± 678.62^#^	5019.70 ± 840.35	0.002^*^

* Indicates statistically differences between groups (*p* < 0.05), # indicates differences compared to the CON (*p* < 0.05), & indicates differences compared to the HYP (*p* < 0.05).

For the change in $\dot{V}O_{2}$ during exercise, differences were observed in the main effect at various time points (F (12, 396) = 323.664, *p* < 0.001, η_p_^2^ = 0.907). There were disparities in group main effects (F (2, 33) = 7.509, *p* = 0.002, η_p_^2^ = 0.313), and differences in time and group interaction effects were also identified (F = 2.790, *p* < 0.001, η_p_^2^ = 0.145). Specifically, compared to the HOT, the HYP and CON showed a increase in $\dot{V}O_{2}$ from the 2nd to the 7th minute (*p* < 0.05). From the 7th to the 9th minute, the HOT exhibited a decrease in $\dot{V}O_{2}$ compared to the CON (*p* < 0.05). At the 10th minute and $\dot{V}O_{2\max}$ , both the HOT and HYP demonstrated a reduction in $\dot{V}O_{2}$ compared to the CON (*p* < 0.05) are shown in Figure 1C.

**FIGURE 2:**
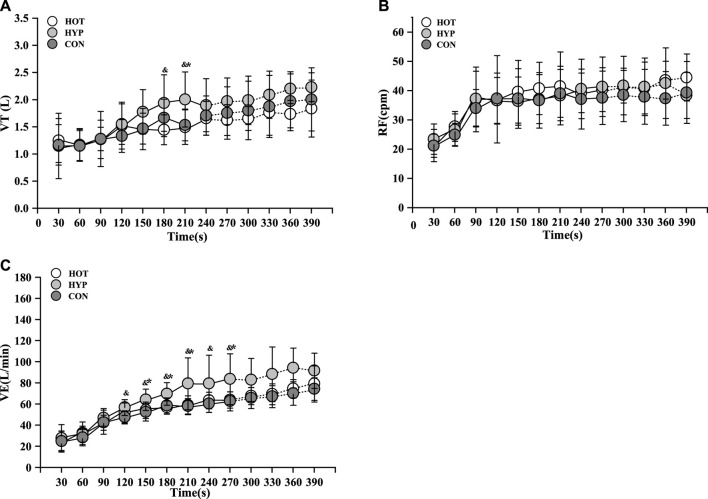
Changes of VT **(A)** and RF **(B)** and VE **(C)** in incremental exhaustive exercise in different environments. Note: * indicates a difference between the CON and HYP (*p* < 0.05), # indicates a difference between the CON and HOT (*p* < 0.05), and & indicates a difference between the HOT and HYP (*p* < 0.05).

For the change in VT during exercise, differences were observed in the main effect at various time points (F (9, 297) = 31.103, *p* < 0.001, η_p_^2^ = 0. 485). There were disparities in group main effects (F (9, 297) = 31.103, *p* < 0.001, η_p_^2^ = 0. 485), and differences in time and group interaction effects were also identified (F = 2.551, *p* = 0.001, η_p_^2^ = 0.134). The results indicated that, in comparison to HYP, CON exhibited a lower VT at 210s (*p* < 0.05), and HOT showed a lower VT at 180s and 210s shown in Figure 2A.

For the change in RF during exercise, differences were observed in the main effect at various time points (F (9, 297) = 35.231, *p* < 0.001, η_p_^2^ = 0.516). There was no discernible difference in the group main effect (F (2, 33) = 1.057, *p* = 0.359, η_p_^2^ = 0.060), and no substantial difference in the time and group interaction effect (F = 1.122, *p* = 0.329, η_p_^2^ = 0.064). While there was no increase in RF within the first 270s for HYP, the RF remained relatively high during this period shown in Figure 2B.

For the change in VE during exercise, differences were observed in the main effect at various time points (F (9, 297) = 150.155, *p* < 0.001, η_p_^2^ = 0.820). There were disparities in group main effects (F (2, 33) = 5.468, *p* = 0.009, η_p_^2^ = 0.249), and differences in time and group interaction effects were also identified (F = 3.901, *p* < 0.001, η_p_^2^ = 0. 191). Additionally, in the comparison of VE, it was observed that CON had a lower VE than HYP from 120s to 270s (*p* < 0.05), and HOT had a lower VE than HYP at 180s, 210s, and 270s (*p* < 0.05) shown in Figure 2C.

### 3.2 Skeletal muscle hemoglobin

**FIGURE 3:**
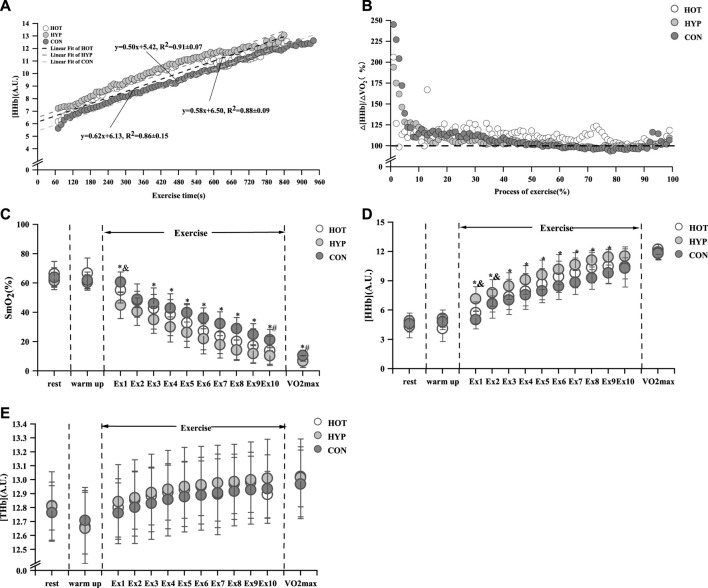
Linear fit of HHb **(A)** and characteristics of △[HHb]/△ $\dot{V}O_{2}$ **(B)** and changes of SmO_2_ **(C)** and HHb **(D)** and THb **(E)** in incremental exhaustive exercise under different environments. Note: * indicates a difference between the CON and HYP (*p* < 0.05), # indicates a difference between the CON and HOT (*p* < 0.05), and & indicates a difference between the HOT and HYP (*p* < 0.05).

The study results indicate that after linear fitting of [HHb] values with exercise time for each group, the HOT had *R* ^2^ = 0.86 ± 0.15 (*p* < 0.01), the HYP had *R* ^2^ = 0.88 ± 0.09 (*p* < 0.01), and the CON had *R* ^2^ = 0.91 ± 0.07 (*p* < 0.01), as shown in Figure 3A. The results show that compared to the CON, the HOT exhibits a increase in △E_HHb_ (F (1.695, 18.643) = 3.796, *p* = 0.047, η_p_^2^ = 0. 257, CON vs. HOT *p* = 0.044 95%CI [0.003, 0.248]). Additionally, the study results reveal that compared to the CON, both the HOT and HYP exhibit a decrease in the exercise time corresponding to BP (F (2, 22) = 4.860, *p* = 0.018, η_p_^2^ = 0.306, CON vs. HOT *p* = 0.049 95%CI [0.004, 2.857]; CON vs. HYP *p* = 0.031, 95%CI [0.144, 2.420]). Furthermore, compared to the CON, the HOT shows a decrease in $\dot{V}O_{2}$ at the BP (F (2, 22) = 7.488, *p* = 0.003, η_p_^2^ = 0. 405, CON vs. HOT *p* = 0.048, 95%CI [5.893, 1173.387]), and both the HOT and HYP exhibit a decrease in relative $\dot{V}O_{2}$ at the BP (F (2, 22) = 8.177, *p* = 0.009, η_p_^2^ = 0.426, CON vs. HOT *p* = 0.035 95%CI [0.517, 15.574]; CON vs. HYP *p* = 0.046, 95%CI [0.088, 10.001]). Subsequently, using BP as a breakpoint, a bilinear fitting was performed on the $\dot{V}O_{2}$ and exercise time before and after BP for each group. The results show that the HOT had R_HHb-1_^2^ = 0.88 ± 0.08 (*p* < 0.01) and R_HHb-2_^2^ = 0.80 ± 0.10 (*p* < 0.01), the HYP had R_HHb-1_^2^ = 0.84 ± 0.10 (*p* < 0.01) and R_HHb-2_^2^ = 0.83 ± 0.22 (*p* < 0.01), and the CON had R_HHb-1_^2^ = 0.87 ± 0.05 (*p* < 0.01) and R_HHb-2_^2^ = 0.87 ± 0.10 (*p* < 0.01). The results also show that compared to the CON, the HYP exhibits a increase in △E_HHb-1_ (F (2, 22) = 4.984, *p* = 0.016, η_p_^2^ = 0.312, CON vs. HOT *p* = 0.005, 95%CI [0.109, 0.566]; CON vs. HYP *p* = 0.029, 95%CI [0.039, 0.782]), as shown in Table 3 Correlations were found between GET and BP in all groups (r_CON_ = 0.635, *P*_CON_ = 0.027; r_HYP_ = 0.872, *P*_CON_<0.001; r_HOT_ = 0.931, *P*_HOT_<0.001).

**TABLE 3:** The dynamic parameter difference of HHb in the incremental exhaustive exercise under different environments.

Variables	HOT	HYP	CON	*p*
Time@BP (min)	6.58 ± 1.08	6.73 ± 0.69	8.01 ± 1.64	0.018^*^
$\dot{V}O_{2}$ @BP (mL/min)	2702.14 ± 391.06^#^	2924.22 ± 385.05	3291.78 ± 606.11	0.003^*^
$\dot{V}O_{2}$ @BP (ml/min·kg)	38.36 ± 5.07^#^	41.36 ± 3.47^#^	46.40 ± 5.38	0.009^*^
*△E*_HHb_ (s^-1^)	0.63 ± 0.13^#^	0.58 ± 0.12	0.50 ± 0.08	0.047^*^
*△E*_HHb-1_ (s^-1^)	0.85 ± 0.33^#^	0.92 ± 0.39^#^	0.51 ± 0.14	0.016^*^
*△E*_HHb-2_ (s^-1^)	0.42 ± 0.23	0.37 ± 0.19	0.48 ± 0.20	0.918

* indicates statistically differences between groups (*p* < 0.05), # indicates differences compared to the CON (*p* < 0.05), and and indicates differences compared to the HYP (*p* < 0.05).

Furthermore, there were differencesand CON reached in $△\left[\text{HHb}\right]/△\dot{V}O_{2}$ during exercise under different conditions. Compared to the HOT, the HYP and CON reached 100% of $△\left[\text{HHb}\right]/△\dot{V}O_{2}$ .as shown in Figure 3B.

After analyzing the data from the first 10min of exercise, For the change in SmO_2_ during exercise, differences were observed in the main effect at various time points (F (12, 396) = 323.664, *p* < 0.001, η_p_^2^ = 0.907). There were disparities in group main effects (F (2, 33) = 7.509, *p* = 0.002, η_p_^2^ = 0.313), and differences in time and group interaction effects were also identified (F = 2.790, *p* < 0.001, η_p_^2^ = 0.145). It was found that in the first minute of incremental load testing, the HOT and CON had a increase in SmO_2_ compared to the HYP (*p* < 0.05). From the 3rd to the 9th minute, the HYP had a decrease in SmO_2_ compared to the CON (*p* < 0.05). At the 10th minute of the incremental load test, the HOT had a decrease in SmO_2_ compared to the CON (*p* < 0.05). When reaching $\dot{V}O_{2\max}$ , both the HOT and HYP had a decrease in SmO_2_ compared to the CON (*p* < 0.05) are shown in Figure 3C. Analyzing the changes in [HHb] during exercise, For the change in [HHb] during exercise, differences were observed in the main effect at various time points (F (12, 396) = 257.178, *p* < 0.001, η_p_^2^ = 0.886). There were disparities in group main effects (F (2, 33) = 6.378, *p* = 0.005, η_p_^2^ = 0.279), and differences in time and group interaction effects were also identified (F = 2.254, *p* < 0.001, η_p_^2^ = 0.120). It was found that from the 1st to the 2nd min, both the CON and HOT had a decrease in [HHb] compared to the HYP (*p* < 0.05). From the 3rd to the 9th minute, the CON had a decrease in [HHb] compared to the HYP (*p* < 0.05) are shown in Figure 3D.

In addition, For the change in [THb] during exercise, differences were observed in the main effect at various time points (F (12, 396) = 31.572, *p* < 0.001, η_p_^2^ = 0.489). There was no discernible difference in the group main effect (F (2, 33) = 0.208, *p* = 0.813, η_p_^2^ = 0.012), and no substantial difference in the time and group interaction effect (F = 0.893, *p* = 0.612, η_p_^2^ = 0.051). we found that no difference in [THb] during exercise among the three environments (*p* > 0.05), as shown in Figure 3E.

**FIGURE 4:**
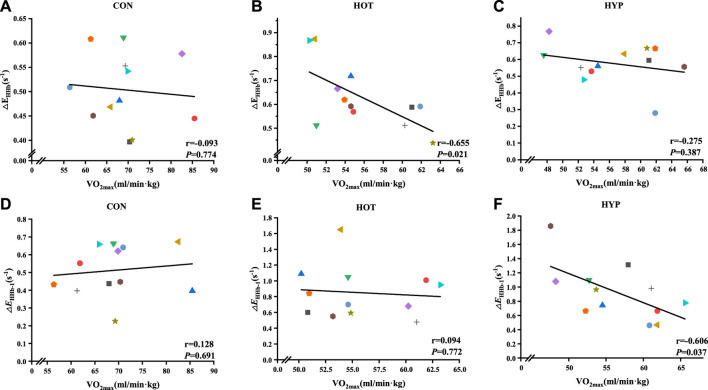
Correlation analysis results for △_EHHb-1_ and $\dot{V}O_{2\max}$ in CON **(A)**, HOT **(B)**, and HYP **(C)**, as well as correlation analysis results for △_EHHb_ and $\dot{V}O_{2\max}$ in CON **(D)**, HOT **(E)**, and HYP **(F)** during incremental exhaustive exercise under different environments.

Additionally, the study analyzed the correlation between △E_HHb_ and corresponding $\dot{V}O_{2\max}$ under different conditions and found a correlation between the two in the HOT (r_HOT_ = −0.655, P_HOT_ = 0.021), as shown in Figures 4A–C. Moreover, the study analyzed the correlation between △E_HHb-1_ and the corresponding $\dot{V}O_{2\max}$ under different conditions, and found a negative correlation bewteen the two in the HYP condition (r_HYP_ = −0.606, PHYP = 0.037), as shown in Figures 4D–F.

## 4 Discussion

The aim of this study was to compare the effects of incremental exhaustive exercise on the dynamic changes of athletes’ $\dot{V}O_{2}$ and HHb under different environments, in order to elucidate the relationship between the dynamic changes of $\dot{V}O_{2}$ and HHb and peripheral fatigue in different environments. Our results demonstrate that compared to normal condition, both hypoxia exposure and heat exposure can lead to a reduction in exhaustion time and an acceleration of fatigue development, thereby negatively impacting aerobic capacity, such as gas exchange and oxygen supply in skeletal muscle tissue. Additionally, the dynamic changes of HHb in different modes indicated that hypoxic and heat stress might individually contribute to the reduction of athletes’ aerobic capacity.

When comparing the dynamic changes of $\dot{V}O_{2}$ during the process of incremental exhaustive exercise under different environments, our study revealed a significant reduction in $\Delta E_{\dot{V}O2}$ and OUES in HOT and HYP. These findings indicate that the gas exchange ability of athletes was inhibited in both environments. Specifically, during exercise in a high-temperature and high humidity environment, the $\dot{V}O_{2}$ between 2 min and 10 min in the HOT was lower compared to the CON, which aligns with the research findings of Sawka (Sawka et al., 1985), González (González-Alonso and Calbet, 2003), and Wingo (Wingo et al., 2020). This can be attributed to the impact of heat stress on the cardiovascular system and oxygen delivery (Cheuvront et al., 2010). Heat stress reduces Cardiac Output (Q) and Mean Arterial Blood Pressure (MAP) by decreasing cardiac output, resulting in reduced skeletal muscle blood flow, oxygen delivery, and $\dot{V}O_{2}$ during exercise. Heat stress can reduce blood flow in skeletal muscles during exercise by enhancing sympathetic nerve activity (Sawka et al., 1985). Therefore, during exercise under heat stress, athletes may reduce their oxygen consumption, which can result in a decrease in $\dot{V}O_{2}$ and $\Delta E_{\dot{V}O2}$ . Additionally, our study observed a significant decrease in $\Delta E_{\dot{V}O2}$ and OUES of athletes under low-oxygen conditions, which aligns with the research findings of Wagner (Wagner et al., 1986) and Loeppky (Loeppky et al., 2020). However, it is important to note that these two studies did not specifically compare the slope of $\dot{V}O_{2}$ before and after the GET. Nevertheless, our study found an increase in $\Delta E_{\dot{V}O2‐1}$ in the hypoxic environment. This finding may be attributed to the fact that the initial stage of exercise in a hypoxic environment effectively stimulates peripheral chemoreceptors, leading to increased depth and acceleration of respiration through reflex mechanisms. Furthermore, the utilization of oxygen reserves in the body results in a decrease in SpO_2_. We believe that one of the reasons for the increase in △E_VO2-1_ is the elevation in VE resulting from enhanced and accelerated respiration due to hypoxic exposure. Firstly, the decrease in oxygen reserve in the body during exercise affects oxygen kinetics. The increased VE under hypoxic conditions improves ventilation efficiency in the early stages of exercise, thereby offsetting the reduction in oxygen reserve (Engelen et al., 1996), resulting in an overall increase in VO_2_ slope with decreasing blood oxygen saturation. Secondly, the elevated VE under hypoxic exposure leads to increased oxygen consumption of respiratory muscles and subsequently increased ventilation cost (Coast et al., 1993; Benoit et al., 1997), thereby influencing VO_2_ production. Consequently, the $\dot{V}O_{2}$ and working rate during the initial stage of exercise increase as the body ingests more oxygen to meet its normal work demands. Therefore, the results of $\dot{V}O_{2}$ in HYP differ from those in HOT, with no significant decrease observed in $\dot{V}O_{2}$ during the initial stage of exercise in HYP. However, as the duration of exercise increases, athletes may experience a decrease in blood perfusion to the respiratory muscles and limitations in gas diffusion. This can result in a mismatch between ventilation and perfusion, ultimately leading to a decrease in $\dot{V}O_{2}$ . Therefore, the response of VE to exercise under hypoxic environment throughout the entire exercise process contributes to the reduction in $\Delta E_{\dot{V}O2}$ (Amann and Calbet, 2008).

Regarding the oxygen supply to skeletal muscles, our findings are consistent with previous literature reports (Zhang et al., 2010), indicating a positive correlation between BP and GET. In terms of selecting the appropriate linear fitting model for HHb, Spencer (Spencer et al., 2012) have noted that the bilinear model provides a more accurate description of the potential physiological response of HHb in subjects compared to the S-type regression model. Thus, we employed the bilinear model to assess the dynamic changes in HHb in VL during exercise. To evaluate the HHb response throughout the entire exercise process, we also calculated the slope of HHb from the onset of exercise to the point of exhaustion. Interestingly, our observations demonstrate that the ΔE_HHb_ in the HOT is higher than that in the CON, and the significance of SmO_2_ is lower in the HOT compared to the CON at the 10-min mark during exercise. Previous research by Dennis (Dennis et al., 2023) has shown that exercise at 35°C and 40°C, compared to a single exercise at 20°C, can enhance the deoxygenation reaction in skeletal muscles. Girard (Girard et al., 2016) have highlighted that high-intensity exercise in a high temperature and humidity environment can affect the efficiency of output power and accelerate peripheral fatigue. Our study indicates that the efficiency of output power of skeletal muscles increases during the initial stage of exercise in the high-temperature and high-humidity environment, offsetting some of the negative effects (Racinais et al., 2017). Consequently, we did not observe a significant decrease in SmO_2_ in the VL during the initial stage of exercise. However, as exercise intensity and duration increased, along with an increase in Tc, peripheral fatigue eventually set in. Throughout the entire exercise process, the significant increase in ΔE_HHb_ may be attributed to the rightward shift of the oxygen dissociation curve caused by a decrease in Hb affinity for oxygen as temperature rises, promoting oxygen release (Webb et al., 2022). Simultaneously, in the analysis of $\dot{V}O_{2}$ and HHb, the $\dot{\Delta V}O_{2}$ /ΔHHb value in the HOT also revealed an enhancement in skeletal muscle Hb deoxygenation reaction. In particular, during the initial stage of exercise, we observed an increase in E_HHb-1_ in the HOT condition compared to the CON condition, while E_VO2-1_ remained unchanged. This observation aligns with the findings of Nybo et al. (Nybo et al., 2001), who reported that heat stress did not influence the oxygen uptake rate in the early stages of exercise but led to a reduction in oxygen uptake and oxygen pulse. It has been suggested that oxygen uptake kinetics are influenced by both the rise in skin temperature and core temperature (Rowell et al., 1966; José et al., 1997), although they do not increase simultaneously during the early stages of exercise. Despite the acceleration of skeletal muscle deoxygenation in these early stages, compensatory mechanisms appear to address the lack of oxygen delivery. Our investigation revealed an increase in HR in the HOT condition, accompanied by an elevation in cardiac output within a certain heart rate range to compensate for the mismatch between oxygen intake and utilization. As heart rate increased within a specific range, cardiac output(Q) also rose to compensate for the discrepancy between oxygen intake and utilization. Additionally, it has been noted that higher Q, hemoconcentration, and enhanced O_2_ extraction contribute to a similar initial rate of rise in $\dot{V}O_{2}$ (González-Alonso and Calbet, 2003). Therefore, the early-stage mismatch between oxygen uptake and utilization in exercise under high temperature and high humidity may result from various factors. Furthermore, the correlation results showed that ΔE_HHb_ in the HOT was negatively correlated with $\dot{V}O_{2\max}$ , suggesting that the increase in Hb deoxygenation reaction during exercise, resulting from the aforementioned mechanism, may be associated with a decrease in exercise duration. Hence, athletes engaging in physical activity within high-temperature, high-humidity environments conditions may potentially influence the interplay between oxygen uptake in skeletal muscles and lungs.

Hypoxia exposure negatively affects athletes during incremental exhaustive exercise, resulting in decreased SpO_2_, SmO_2_, and VO_2max_ at the point of exhaustion. This is consistent with findings from previous studies (Osawa et al., 2011; Azevedo et al., 2020a). Osawa (Osawa et al., 2017) and Bowen (Bowen et al., 2016) have also demonstrated that the SmO_2_ curve of skeletal muscle decreases while the HHb curve increases during incremental exhaustive exercise in a hypoxic environment. However, these studies did not compare the slope of HHb during exercise. In the initial stage of incremental exhaustive exercise in a hypoxic environment, the metabolism is immediately affected. Hypoxia exposure causes the anaerobic energy supply system to be utilized earlier to maintain ATP demand (Linnarsson et al., 1974), leading to a left shift in BP and GET. Previous studies have shown that the value of ΔE_HHb_ during incremental exhaustive exercise can be influenced by factors such as body position (DiMenna et al., 2010) and metabolic diseases (Gildea et al., 2019). In our study, we observed a significant increase in ΔE_HHb-1_ before BP, indicating that as skeletal muscle deoxygenation accelerates, the ability to increase peripheral oxygen delivery and meet the increased oxygen demand decreases. However, in the study by Azevedo (Azevedo et al., 2020b), only the leftward shift of BP and the increase in HHb during exercise under hypoxia were observed, without affecting ΔE_HHb-1_. In comparison, our study employed a lower FiO_2_ for exercise, which may explain the enhanced skeletal muscle deoxygenation response. Acute hypoxia exposure leads to a significant reduction in oxygen delivery to skeletal muscles during exercise, but this can be compensated by increased oxygen uptake in the body (Calbet et al., 2009). The increase in $\Delta E_{\dot{V}O2‐1}$ and $\Delta \dot{V}O_{2}$ /ΔHHb before GET reflects the relationship between the decrease in oxygen reserve and the increase in oxygen uptake. The earlier deployment of the anaerobic energy supply system causes the slope of HHb to rise rapidly in the initial stage, and impaired hemodynamic response can be considered a potential mechanism for decreased exercise capacity (Gildea et al., 2019). Additionally, the decrease in muscle O_2_ flow may potentially limit the possibility of $\dot{V}O_{2}$ kinetics during exercise (Koga et al., 2007). The observed correlation between ΔE_HHb-1_ and $\dot{V}O_{2\max}$ in the HYP group supports this perspective.

In our study, we also observed that ΔE_HHb-2_ in the HHb plateau after BP appeared to be unaffected by the environmental factors, supporting the notion that the increase in HHb near the critical exercise intensity is not limited by the oxygen diffusion capacity (Murias et al., 2013). Iannetta (Iannetta et al., 2018) have indicated that an oxygen reserve can still be observed in the deep layer of the VL during incremental exhaustive exercise, and this is not influenced by gender or training level (Inglis et al., 2019). Therefore, the presence of an HHb plateau does not indicate the upper limit of oxygen extraction. After reaching BP, the diffusion capacity of oxygen from the capillaries to the muscle fibers may have reached its peak, and the subsequent increase in oxygen uptake depends more on increased oxygen delivery. In our study, we did not observe any difference in $\Delta E_{\dot{V}O2‐2}$ among the three environments, which consequently led to no difference in ΔE_HHb-2_. This suggests that the oxygen extraction capacity during the HHb plateau phase is not influenced by the environmental conditions.

Some criticisms persist regarding the use of near-infrared spectroscopy in the determination of tissue oxygenation. NIRS signals capture changes in hemoglobin Hb and Mb oxygenation, enabling a robust assessment of muscle oxygenation status throughout all exercise stages with high precision (Lucero et al., 2018). Muscle oxygenation reflects the equilibrium between oxygen delivery and oxygen utilization (Koga et al., 2007). However, the contributions of Hb and Mb to NIRS signals differ during muscle contraction (Spires et al., 2011). Other factors, including hematocrit, blood volume, arterioles, capillaries, and venous distribution, can also influence the interpretation of NIRS dynamics in oxygenation and deoxygenation (Koirala et al., 2021). With increased blood flow during exercise, THb may be affected (Alvares et al., 2020). However, even when skin and muscle blood flow increase simultaneously, changes in NIRS-derived oxygenation signals (SmO_2_, HHb) can still accurately reflect alterations in muscle oxygenation (Tew et al., 2010). Some studies have suggested that the oxygenated signal is influenced by increased skin blood flow, while the deoxygenation signal is not sensitive to changes in blood volume (Grassi et al., 2003; Grassi and Quaresima, 2016). Additionally, Koirala et al.'s study (Koirala et al., 2021) found that changes in blood volume have an additional impact on oxygenation Hb-Mb, primarily associated with capillary oxygenation Hb. In contrast, the effect on deoxidation HHb-Mb is less pronounced, given its association with abundant O_2_ delivery due to the high O_2_ saturation of Hb and Mb. Therefore, based on the aforementioned evidence, we maintain confidence in exploring deoxyhemoglobin dynamics in athletes during increasing load exercises under different environments.

## 5 Conclusion

When athletes engage in incremental exhaustive exercise in a hypoxic environment or a high temperature and high humidity environment, the gas exchange in the body and the oxygen supply to skeletal muscle tissue can be compromised. This can have implications for athletes, as accelerated deoxygenation of skeletal muscle during increasing load exercise under high temperature and high humidity, and excessive deoxygenation of skeletal muscle before the break point of deoxygenated hemoglobin under hypoxic environments, may contribute to peripheral fatigue in different conditions.

In high temperature and high humidity environments, exercise intensity can negatively impact the skeletal muscle deoxygenation response. On the other hand, low to moderate load training in a hypoxic environment can accelerate the skeletal muscle deoxygenation response. Therefore, coaches should take into account the specific characteristics of peripheral fatigue during training or competition in different environments and design appropriate training or competition programs accordingly. It is important to consider the limitations imposed by these environments and develop strategies to optimize performance and mitigate the negative effects of reduced oxygen availability and increased heat and humidity.

## Figures and Tables

**Table:** 

**△E**_**HHb**_	Linear fitting slope of HHb
**△E**_**HHb-1**_	Linear fitting slope of HHb before BP
**△E**_**HHb-2**_	Linear fitting slope of HHb after BP
$△E_{\dot{V}O2}$	Linear fitting slope of $\dot{V}O_{2}$
$△E_{\dot{V}O2‐1}$	Linear fitting slope of $\dot{V}O_{2}$ before GET
$△E_{\dot{V}O2‐2}$	Linear fitting slope of $\dot{V}O_{2}$ after GET
**ANOVA**	Analysis of variance
**Bla**	Blood lactate
**BP**	Break point
**CON**	Normal temperature and humidity with normoxia
**FiO**_**2**_	Fractional inspired oxygen concentration
**GET**	Gas exchange threshold
**HHb**	Deoxyhemoglobin
**HOT**	High temperature and humidity with normoxia
**HR**	Heart rate
**HYP**	Normal temperature and humidity with hypoxia
**Mb**	Myoglobin
**NIRS**	Near-infrared spectroscopy
**OUES**	Oxygen uptake efficiency slope
**RCP**	Respiratory compensation point
**RER**	respiratory exchange ratio
**RF**	Respiratory frequency
**RH**	Relative humidity
**RPE**	Borg rating of perceived exertion
**SD**	Standard division
**SmO**_**2**_	Muscle oxygen saturation
**SpO**_**2**_	Oxygen saturation
**Tc**	Core temperature
**THb**	Total hemoglobin
$\dot{V}{\text{CO}}_{2}$	Carbon dioxide production
**VE**	Minute ventilation
**VL**	Vastus lateralis muscles
$\dot{V}O_{2}$	Oxygen uptake
$\dot{V}O_{2\max}$	Maximal oxygen uptake
**VT**	Tidal volume

## Data Availability

The raw data supporting the conclusion of this article will be made available by the authors, without undue reservation.

## References

[B1] Alvares T. S. Oliveira G. V. D. Soares R. Murias J. M. (2020). Near-infrared spectroscopy-derived total haemoglobin as an indicator of changes in muscle blood flow during exercise-induced hyperaemia. J. sports Sci. 38, 751–758. 10.1080/02640414.2020.1733774 32106780

[B2] Amann M. Calbet J. (2008). Convective oxygen transport and fatigue. J. Appl. Physiology 104, 861–870. 10.1152/japplphysiol.01008.2007 17962570

[B3] Azevedo R. D. Je B. S. Inglis E. C. Iannetta D. Murias J. M. (2020a). Hypoxia equally reduces the respiratory compensation point and the NIRS‐derived [HHb] breakpoint during a ramp‐incremental test in young active males. Physiol. Rep. 8, e14478. 10.14814/phy2.14478 32592338 PMC7319946

[B4] Azevedo R. D. A. BéJAR Saona J. E. Inglis E. C. Iannetta D. Murias J. M. (2020b). The effect of the fraction of inspired oxygen on the NIRS-derived deoxygenated hemoglobin “breakpoint” during ramp-incremental test. Am. J. Physiology-Regulatory, Integr. Comp. Physiol. 318, R399–R409. 10.1152/ajpregu.00291.2019 PMC7052603 31850819

[B5] Beaver W. L. Wasserman K. Whipp B. J. (1986). A new method for detecting anaerobic threshold by gas exchange. J. Appl. Physiol. 60, 2020–2027. 10.1152/jappl.1986.60.6.2020 3087938

[B6] Benoit H. Busso T. Prieur F. Castells J. Freyssenet D. Lacour J. R. (1997). Oxygen uptake during submaximal incremental and constant work load exercises in hypoxia. Int. J. sports Med. 18, 101–105. 10.1055/s-2007-972603 9081265

[B7] Boone J. Barstow T. J. Celie B. Prieur F. Bourgois J. (2015). The impact of pedal rate on muscle oxygenation, muscle activation and whole-body VO₂ during ramp exercise in healthy subjects. Eur. J. Appl. Physiol. 115, 57–70. 10.1007/s00421-014-2991-x 25204279

[B8] Boushel R. Langberg H. Olesen J. Gonzales-Alonzo J. Bülow J. Kjaer M. (2001). Monitoring tissue oxygen availability with near infrared spectroscopy (NIRS) in health and disease. Scand. J. Med. Sci. Sports 11, 213–222. 10.1034/j.1600-0838.2001.110404.x 11476426

[B9] Bowen T. S. Koga S. Amano T. Kondo N. Rossiter H. B. (2016). The spatial distribution of absolute skeletal muscle deoxygenation during ramp-incremental exercise is not influenced by hypoxia. Adv. Exp. Med. Biol. 876, 19–26. 10.1007/978-1-4939-3023-4_2 26782190

[B10] Calbet J. A. RåDEGRAN G. Boushel R. Saltin B. (2009). On the mechanisms that limit oxygen uptake during exercise in acute and chronic hypoxia: role of muscle mass. J. Physiol. 587, 477–490. 10.1113/jphysiol.2008.162271 19047206 PMC2670057

[B11] Chance B. Dait M. T. Zhang C. Hamaoka T. Hagerman F. (1992). Recovery from exercise-induced desaturation in the quadriceps muscles of elite competitive rowers. Am. J. Physiology-Cell Physiol. 262, C766–C775. 10.1152/ajpcell.1992.262.3.C766 1312785

[B12] Cheuvront S. N. Kenefick R. W. Montain S. J. Sawka M. N. (2010). Mechanisms of aerobic performance impairment with heat stress and dehydration. J. Appl. Physiol. 109, 1989–1995. 10.1152/japplphysiol.00367.2010 20689090

[B13] Coast J. Rasmussen S. Krause K. M. O'Kroy J. A. Loy R. A. Rhodes J. (1993). Ventilatory work and oxygen consumption during exercise and hyperventilation. J. Appl. Physiol. 74, 793–798. 10.1152/jappl.1993.74.2.793 8458797

[B14] Crum E. O’Connor W. Van Loo L. Valckx M. Stannard S. R. (2017). Validity and reliability of the Moxy oxygen monitor during incremental cycling exercise. Eur. J. Sport Sci. 17, 1037–1043. 10.1080/17461391.2017.1330899 28557670

[B15] Dennis M. C. Goods P. S. Binnie M. J. Girard O. Wallman K. E. Dawson B. (2023). Increased air temperature during repeated-sprint training in hypoxia amplifies changes in muscle oxygenation without decreasing cycling performance. Eur. J. Sport Sci. 23, 62–72. 10.1080/17461391.2021.2003868 34743674

[B16] Dimenna F. J. Bailey S. J. Jones A. M. (2010). Influence of body position on muscle deoxy [Hb+ Mb] during ramp cycle exercise. Respir. physiology Neurobiol. 173, 138–145. 10.1016/j.resp.2010.07.005 20654739

[B17] Engelen M. Porszasz J. Riley M. Wasserman K. Maehara K. Barstow T. J. (1996). Effects of hypoxic hypoxia on O2 uptake and heart rate kinetics during heavy exercise. J. Appl. Physiol. 81, 2500–2508. 10.1152/jappl.1996.81.6.2500 9018498

[B18] Gildea N. Rocha J. Mcdermott A. O'Shea D. Green S. Egaña M. (2019). Influence of type 2 diabetes on muscle deoxygenation during ramp incremental cycle exercise. Respir. Physiology Neurobiol. 269, 103258. 10.1016/j.resp.2019.103258 31349019

[B19] Girard O. Brocherie F. Morin J.-B. Millet G. P. (2016). Running mechanical alterations during repeated treadmill sprints in hot versus hypoxic environments. A pilot study. J. sports Sci. 34, 1190–1198. 10.1080/02640414.2015.1101482 26473996

[B20] GonzáLEZ-Alonso J. Calbet J. A. (2003). Reductions in systemic and skeletal muscle blood flow and oxygen delivery limit maximal aerobic capacity in humans. Circulation 107, 824–830. 10.1161/01.cir.0000049746.29175.3f 12591751

[B21] Grassi B. Pogliaghi S. Rampichini S. Quaresima V. Ferrari M. Marconi C. (2003). Muscle oxygenation and pulmonary gas exchange kinetics during cycling exercise on-transitions in humans. J. Appl. Physiol. 95, 149–158. 10.1152/japplphysiol.00695.2002 12611769

[B22] Grassi B. Quaresima V. (2016). Near-infrared spectroscopy and skeletal muscle oxidative function *in vivo* in health and disease: a review from an exercise physiology perspective. J. Biomed. Opt. 21, 091313. 10.1117/1.JBO.21.9.091313 27443955

[B23] Iannetta D. Okushima D. Inglis E. C. Kondo N. Murias J. M. Koga S. (2018). Blood flow occlusion-related O2 extraction “reserve” is present in different muscles of the quadriceps but greater in deeper regions after ramp-incremental test. J. Appl. Physiol. 125, 313–319. 10.1152/japplphysiol.00154.2018 29722622

[B24] Inglis E. C. Iannetta D. Murias J. M. (2019). Evaluating the NIRS-derived microvascular O2 extraction “reserve” in groups varying in sex and training status using leg blood flow occlusions. Plos One 14, e0220192. 10.1371/journal.pone.0220192 31344091 PMC6658081

[B25] José G. L.-A. Mora-Rodriguez R. Below P. R. Coyle E. F. (1997). Dehydration markedly impairs cardiovascular function in hyperthermic endurance athletes during exercise. J. Appl. Physiol. 82, 1229–1236. 10.1152/jappl.1997.82.4.1229 9104860

[B26] Jung W.-S. Kim S.-W. Park H.-Y. Kim J. Lim K. (2021). Effects of acute exposure to thermal stress on cardiorespiratory function, skeletal muscle oxygenation, and exercise performance in healthy males. Int. J. Environ. Res. Public Health 18, 7404. 10.3390/ijerph18147404 34299853 PMC8307583

[B27] Koga S. Poole D. C. Ferreira L. F. Whipp B. J. Kondo N. Saitoh T. (2007). Spatial heterogeneity of quadriceps muscle deoxygenation kinetics during cycle exercise. J. Appl. Physiol. 103, 2049–2056. 10.1152/japplphysiol.00627.2007 17885024

[B28] Koirala B. Concas A. Sun Y. Gladden L. B. Lai N. (2021). Blood volume versus deoxygenated NIRS signal: computational analysis of the effects muscle O2 delivery and blood volume on the NIRS signals. J. Appl. Physiol. 131, 1418–1431. 10.1152/japplphysiol.00105.2021 34528461 PMC8906537

[B29] Lawler J. S. Powers S. K. Thompson D. (1988). Linear relationship between VO2max and VO2max decrement during exposure to acute hypoxia. J. Appl. Physiol. 64, 1486–1492. 10.1152/jappl.1988.64.4.1486 3378983

[B30] Linnarsson D. Karlsson J. Fagraeus L. Saltin B. (1974). Muscle metabolites and oxygen deficit with exercise in hypoxia and hyperoxia. J. Appl. Physiol. 36, 399–402. 10.1152/jappl.1974.36.4.399 4820319

[B31] Loeppky J. Salgado R. Sheard A. Kuethe D. O. Mermier C. M. (2020). Variations in exercise ventilation in hypoxia will affect oxygen uptake. Physiol. Int. 107, 431–443. 10.1556/2060.2020.00031 33021952

[B32] Lucero A. A. Addae G. Lawrence W. Neway B. Credeur D. P. Faulkner J. (2018). Reliability of muscle blood flow and oxygen consumption response from exercise using near‐infrared spectroscopy. Exp. Physiol. 103, 90–100. 10.1113/EP086537 29034529 PMC12884174

[B33] Murias J. M. Spencer M. D. Keir D. A. Paterson D. H. (2013). Systemic and vastus lateralis muscle blood flow and O2 extraction during ramp incremental cycle exercise. Am. J. Physiology-Regulatory, Integr. Comp. Physiol. 304, R720–R725. 10.1152/ajpregu.00016.2013 PMC3652075 23515617

[B34] Murias J. M. Spencer M. D. Paterson D. H. (2014). The critical role of O2 provision in the dynamic adjustment of oxidative phosphorylation. Exerc. Sport Sci. Rev. 42, 4–11. 10.1249/JES.0000000000000005 24188979

[B35] Nybo L. Jensen T. Nielsen B. González-Alonso J. (2001). Effects of marked hyperthermia with and without dehydration onV o 2 kinetics during intense exercise. J. Appl. Physiol. 90, 1057–1064. 10.1152/jappl.2001.90.3.1057 11181620

[B36] Osawa T. Arimitsu T. Takahashi H. (2017). Hypoxia affects tissue oxygenation differently in the thigh and calf muscles during incremental running. Eur. J. Appl. Physiol. 117, 2057–2064. 10.1007/s00421-017-3696-8 28819691

[B37] Osawa T. Kime R. Hamaoka T. Katsumura T. Yamamoto M. (2011). Attenuation of muscle deoxygenation precedes EMG threshold in normoxia and hypoxia. Med. Sci. Sports Exerc. 43, 1406–1413. 10.1249/MSS.0b013e3182100261 21266933

[B38] PéRIARD J. D. Thompson M. W. Caillaud C. Quaresima V. (2013). Influence of heat stress and exercise intensity on vastus lateralis muscle and prefrontal cortex oxygenation. Eur. J. Appl. Physiol. 113, 211–222. 10.1007/s00421-012-2427-4 22648526

[B39] Racinais S. Cocking S. PéRIARD J. D. (2017). Sports and environmental temperature: from warming-up to heating-up. Temperature 4, 227–257. 10.1080/23328940.2017.1356427 PMC5605167 28944269

[B40] Rowell L. B. Marx H. J. Bruce R. A. Conn R. D. Kusumi F. (1966). Reductions in cardiac output, central blood volume, and stroke volume with thermal stress in normal men during exercise. J. Clin. Investigation 45, 1801–1816. 10.1172/JCI105484 PMC292862 5926447

[B41] Saitoh T. Ooue A. Kondo N. Niizeki K. Koga S. (2010). Active muscle oxygenation dynamics measured during high-intensity exercise by using two near-infrared spectroscopy methods. Adv. Exp. Med. Biol. 662, 225–230. 10.1007/978-1-4419-1241-1_32 20204796

[B42] Sawka M. N. Leon L. R. Montain S. J. Sonna L. A. (2011). Integrated physiological mechanisms of exercise performance, adaptation, and maladaptation to heat stress. Compr. Physiol. 1, 1883–1928. 10.1002/cphy.c100082 23733692

[B43] Sawka M. N. Young A. J. Cadarette B. S. Levine L. Pandolf K. B. (1985). Influence of heat stress and acclimation on maximal aerobic power. Eur. J. Appl. physiology Occup. Physiol. 53, 294–298. 10.1007/BF00422841 4039255

[B44] Seiyama A. Hazeki O. Tamura M. (1988). Noninvasive quantitative analysis of blood oxygenation in rat skeletal muscle. J. Biochem. 103, 419–424. 10.1093/oxfordjournals.jbchem.a122285 3391996

[B45] Spencer M. D. Murias J. M. Paterson D. H. (2012). Characterizing the profile of muscle deoxygenation during ramp incremental exercise in young men. Eur. J. Appl. Physiol. 112, 3349–3360. 10.1007/s00421-012-2323-y 22270488

[B46] Spires J. Lai N. Zhou H. Saidel G. M. (2011). Hemoglobin and myoglobin contributions to skeletal muscle oxygenation in response to exercise. Adv. Exp. Med. Biol. 701, 347–352. 10.1007/978-1-4419-7756-4_47 21445808 PMC3893190

[B47] Subudhi A. W. Dimmen A. C. Roach R. C. (2007). Effects of acute hypoxia on cerebral and muscle oxygenation during incremental exercise. J. Appl. Physiol. 103, 177–183. 10.1152/japplphysiol.01460.2006 17431082

[B48] Tatterson A. J. Hahn A. G. Martini D. T. Febbraio M. A. (2000). Effects of heat stress on physiological responses and exercise performance in elite cyclists. J. Sci. Med. Sport 3, 186–193. 10.1016/s1440-2440(00)80080-8 11104310

[B49] Tew G. A. Ruddock A. D. Saxton J. M. (2010). Skin blood flow differentially affects near-infrared spectroscopy-derived measures of muscle oxygen saturation and blood volume at rest and during dynamic leg exercise. Eur. J. Appl. physiology 110, 1083–1089. 10.1007/s00421-010-1596-2 20700602

[B50] Twomey R. Wrightson J. Fletcher H. Avraam S. Ross E. Dekerle J. (2017). Exercise-induced fatigue in severe hypoxia after an intermittent hypoxic protocol. Med. Sci. Sports Exerc. 49, 2422–2432. 10.1249/mss.0000000000001371 28708702

[B51] Vieth E. (1989). Fitting piecewise linear regression functions to biological responses. J. Appl. Physiol. 67, 390–396. 10.1152/jappl.1989.67.1.390 2759968

[B52] Wagner P. D. Gale G. E. Moon R. E. Torre-Bueno J. R. Stolp B. W. Saltzman H. A. (1986). Pulmonary gas exchange in humans exercising at sea level and simulated altitude. J. Appl. Physiol. 61, 260–270. 10.1152/jappl.1986.61.1.260 3090012

[B53] Webb K. L. Dominelli P. B. Baker S. E. Klassen S. A. Joyner M. J. Senefeld J. W. (2022). Influence of high hemoglobin-oxygen affinity on humans during hypoxia. Front. Physiol. 12, 763933. 10.3389/fphys.2021.763933 35095551 PMC8795792

[B54] Whipp B. J. Davis J. A. Wasserman K. (1989). Ventilatory control of the ‘isocapnic buffering’region in rapidly-incremental exercise. Respir. Physiol. 76, 357–367. 10.1016/0034-5687(89)90076-5 2501844

[B55] Wingo J. E. Stone T. Ng J. (2020). Cardiovascular drift and maximal oxygen uptake during running and cycling in the heat. Med. Sci. Sports Exerc. 52, 1924–1932. 10.1249/MSS.0000000000002324 32102057

[B56] Yamaguchi K. Sumi D. Hayashi N. Ienaga K. Goto K. (2021). Effects of combined hot and hypoxic conditions on muscle blood flow and muscle oxygenation during repeated cycling sprints. Eur. J. Appl. Physiol. 121, 2869–2878. 10.1007/s00421-021-04738-w 34195866

[B57] Zerbini L. Spencer M. D. Grey T. M. Murias J. M. Kowalchuk J. M. Schena F. (2013). Effect of acute hypoxia on muscle blood flow, VO₂p, and [HHb] kinetics during leg extension exercise in older men. Eur. J. Appl. Physiol. 113, 1685–1694. 10.1007/s00421-013-2599-6 23381722

[B58] Zhang Z. Wang B. Gong H. Xu G. Nioka S. Chance B. (2010). Comparisons of muscle oxygenation changes between arm and leg muscles during incremental rowing exercise with near-infrared spectroscopy. J. Biomed. Opt. 15, 017007. 10.1117/1.3309741 20210481

